# Extent of Home Delivery among Women Who Gave Birth in the Last One Year in Serbo, Kersa Woreda, Jimma Zone, Oromia Region, Southwest Ethiopia

**DOI:** 10.1155/2022/7728127

**Published:** 2022-01-17

**Authors:** Gemechu Terefe, Ahmedin Teha, Tujuba Diribsa, Daba Abdisa

**Affiliations:** ^**1**^ School of Midwifery, Institute of Health, Jimma University, Jimma, Ethiopia; ^2^Department of Reproductive Health Institute of Health, Jimma University, Jimma, Ethiopia; ^3^Department of Biomedical Science, Health Institute of Health, Jimma University, Jimma, Ethiopia

## Abstract

**Background:**

Home delivery is childbirth in a nonclinical setting that takes place in a residence rather than in a health institution. Maternal morbidity and mortality are global health challenges, and developing countries contribute to most of the maternal deaths.

**Objective:**

This study aimed to assess the extent and associated factors for home delivery in Serbo, Kersa Woreda, Jimma Zone, Southwest Ethiopia.

**Method:**

A community-based cross-sectional study was employed among the 240 study participants. Data were collected by using systematic sampling technique from July 5 to 26, 2021, via a pretested semistructured questionnaire through face-to-face interview, and analyzed by a statistical package for the social sciences version 23.0. Bivariable and multivariable logistic regression analyses were carried out to identify factors associated with the extent of home delivery, and factors associated with the extent of home delivery were declared at a *p* value <0.05.

**Result:**

In this study, the extent of home delivery was 28.7%. Identified factors statically associated with home delivery were low monthly income (AOR = 16.7, 95% CI: (2.028–13,83)), only the husband as the decision-maker (AOR = 5.0, 95% CI: (1.252–20.021)), never had a history of ANC follow-up (AOR = 5.7, 95% CI: (2.358–16.3)), poor knowledge toward delivery service (AOR = 3.0, 95% CI: (1.661–5.393)), negative attitude toward delivery service (AOR = 2.2, 95% CI: (1.054–4.409)), and large family size (AOR = 2.2, 95% CI: (1.187–4,119)).

**Conclusion:**

When compared to the Ethiopian Demographic and Health Survey 2016, the prevalence of home delivery among women who gave birth in the last one year was low in this study. The study participants' identified factors that were significantly linked with home delivery were low monthly income, only husband as decision maker, no ANC follow-up, poor knowledge of delivery services, negative attitude toward delivery services, and large family size. Health professionals and health extension workers should raise awareness about institutional delivery and birth readiness so that women can give birth at a health facility even if labor begins unexpectedly.

## 1. Introduction

Childbirth in a nonclinical setting, such as at home rather than in a health facility, is known as home delivery. Between 1990 and 2015, a total of 13.6 million women died from maternal causes around the world [1]. The majority of maternal health difficulties and deaths occurred in low- and middle-income nations, with direct obstetric complications accounting for 75% of deaths [2]. In sub-Saharan Africa, a woman's lifetime risk of dying from treatable or preventable pregnancy and delivery problems is 1 in 22, compared to 1 in 7,300 in developed nations [3].

Even though Ethiopia's maternal mortality rate has decreased since the 2016 Ethiopian Demographic and Health Survey (EDHS), it remains one of the highest, with 412 deaths per 100,000 live births [4]. The majority of maternal deaths occur on the first day following delivery, emphasizing the need of receiving high-quality care throughout labor and delivery. The majority of maternal deaths occur as a result of obstetric complications that could have been prevented with adequate medical care by skilled attendants during and after delivery [5].

The impact of home birth, particularly unattended birth, is not confined to maternal health issues; it also leads to perinatal and neonatal morbidity and mortality [6]. Ethiopia was a country where only a small percentage of reproductive-age women sought professional help during pregnancy and childbirth [7].

One-third of all deliveries take place at home without the help of experienced attendants around the world, and in Africa, skilled health workers attend less than half of all births [8]. However, in developing nations, the majority of births take place at home without the assistance of trained birth attendants [3,9]. Regardless of many developing countries' working hard to improve maternal health by optimizing critical and effective maternal health measures, the rate of success is low [10].

Moreover, according to the most recent Ethiopian demographic and health survey, just 26% of births take place in a health facility, with the remaining 74% taking place at home [4]. Only 24.2% of births in Oromia take place in a health facility, with the remaining 75% taking place at home [9]. According to a study finding in Hadiya Zone, 73.6% of women who gave birth in the previous 12 months delivered their babies at home, almost identical to the national figure [11]. If a home delivery is not performed by a trained professional, it raises the risk of infection, postpartum hemorrhage (PPH), and HIV/AIDS transmission to family or traditional birth attendants who do not use protective equipment [12].

There is only one health center in Serbo. The extent of home delivery and the factors that influence it are unknown. As a result, this study was aimed to determine the extent and associated factors for home delivery in Serbo, Kersa Woreda, Jimma Zone, Southwest Ethiopia.

## 2. Methods and Materials

### 2.1. Study Design and Setting

A community-based cross-sectional study design was conducted in Serbo, Kersa Woreda, from July 5 to 26, 2021. Serbo was found in Kersa Woreda, Jimma Zone, Oromia Region, Southwest Ethiopia 22 km north east of Jimma town in the way to Addis Ababa. The total population of Serbo was estimated to be 4206 with a male to female ration of 1.1 among the female population, 40% of whom were found in the reproductive-age group (15–49 years of age) and had a total of 873 households. The means of livelihood of Serbo was mainly mixed type (such as agriculture and animal husbandry). All women who lived in Serbo and gave birth in the last one year were the source of the population. And all systematically selected women who gave birth in the last one year were included in the study population. All women who lived in Serbo who gave birth in the last one-year preceding the study period and were willing to participate were eligible for the study. The sample size was calculated by using the single population proportion formula with the following assumptions: *Z* = the standard normal deviation at 95% confidence interval = 1.96, 50% maximum sample size, and *d* = margin of error that can be tolerated at 5% (0.05). Since the total number of women who gave birth in the last one year was less than 10,000, the sample size was adjusted by using the finite population correction formula. Then, after considering a nonresponse rate of 5%, the final sample size became 266. Every 3 household was selected by systematic sampling technique, and the first house hold was selected by a lottery method. The total number of women who gave birth in the Serbo from December 2019 up to December 2020 the data was taken from registration of women who gave birth in the last one year, from local health extension workers.

### 2.2. Data Collection Measurement and Procedures

Semistructured questionnaires were adapted from literature [13–16]. Questionnaires were first prepared in English version and then translated into local language Afan, Oromo, and Amharic versions to obtain clarified data from the study participants. The questionnaires have 5 five parts to assess sociodemographic characteristics of women, women's knowledge of institutional delivery, women's attitude toward institutional delivery, obstetric factors of women, and the practice of place of delivery. Data were collected by three trained midwives by using a pretested structured questionnaire. All data collectors were wearing masks and kept a social distance during the interviews since it is the time of Covid-19 pandemic. The principal investigator supervises, guides, and facilitates the data collection process. Closed houses were visited two times during the time of data collection and they proceed to the immediate next house. Before the start of data collection, training was offered to data collectors for one day on the technique of data collection, the purpose of data collection, and the content of the questionnaires, as well as on how to approach the respondents and how to deal with difficulties that might arise during the data collection period. A pretest was carried out in Asendabo by taking 5% (13) of the total sample size before one week of actual data collection to assess the instrument. Then, the necessary modifications were undertaken. The principal investigator supervised the ongoing process during the data collection to ensure the quality of the data by checking field format for completeness and consistency.

### 2.3. Data Analysis

Data were entered into EpiData version 4.2 and then exported to SPSS version 21 for analysis. Descriptive statistics such as frequencies, proportions, and percentages were carried out for the categorical variables, while measures of central tendency and dispersion were summarized for continuous data. Bivariate logistic regression was carried out to select a candidate for multivariate logistic regression analysis with *p* value <0.25. Then, candidate variables were entered into multiple logistic regression models using the backward likelihood ratio method to identify the statistically significant factors of home delivery and to control the possible confounders. The model of fitness was checked before multivariable analysis was carried out by using the Hosmer–Lemeshow test which was found to be insignificant (*p* value = 0.147) and the Omnibus test was significant (*p* value = 0.0001). The degree of association between independent and dependent variables were assessed using the odds ratio, and statistically significant factors were declared at 95% of confidence interval and *p* value of less than 0.05.

## 3. Results

### 3.1. Description of Participants

A total of 266 parents were included in this study, of which 26 of them were excluded because of data incompleteness. So, the analysis was based on 240 participants with a response rate of 90.2%.

The mean age of the respondents was 27.23 ± 4.186 SD. The majority of study participants were found in the age range between 25 and 29 years old, making a total of one hundred and eleven participants (46.3%). Regarding the marital status of the study participants, two hundred twenty-three (92.9%) were married, and about 142 (59.2%) were Muslims. Concerning the occupational status of women, one hundred fifteen (47.9%) were housewives. More than half the participants attended primary education (55.4%) (Table 1).

### 3.2. Maternal and Obstetric Characteristics of Women

Concerning the obstetric characteristics, the vast majority of study participants (95%) had never experienced an abortion and lost a child (99.2%), respectively. During their last pregnancy, almost three-fourths (74.6%) of study participants received a follow-up ANC, and 160 (91.1%) received counseling on the birthing location and possible pregnancy and delivery issues. Concerning the time of starting ANC follow-up, the majority of women (92.2%) started in the first trimester, and 75.8% were aware of at least one advantage of the follow-up. From the respondents who did not follow-up ANC, most of them said that they did not have any health problem concerns and were too busy to go, 42.6% and 39.3%, respectively. Regarding obstetric danger signs, about 10% encountered any type of obstetric danger sign. One hundred-sixty (91.1%) received information on the delivery place and possible pregnancy and delivery complications during their last pregnancy's ANC flows (Table 2).

### 3.3. Knowledge Factor toward Institutional Delivery

**Figure 1:**
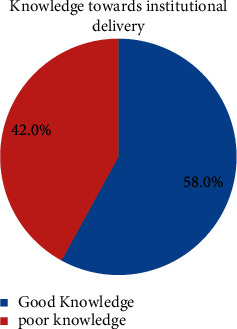
Knowledge of women toward institutional delivery among women who gave birth in the last one year in Serbo, Kersa district, Southwest Ethiopia, July 2021.

Regarding their knowledge factor toward home delivery, 48% of the women knew the date that the baby was expected to arrive. All study participants know the advantage of institutional delivery to prevent complications during delivery and postpartum, including newborn complications. Regarding their knowledge factor toward institutional delivery, 58% of study participants had good knowledge toward institutional delivery and 42% had poor knowledge (Figure 1).

### 3.4. Attitude Factors toward Institutional Delivery

In regard to their attitude about pregnancy and childbirth complications as well as the importance of getting skilled help at childbirth, 66.7% of the women feel that they may be susceptible to developing delivery complications, 62.5% of them perceive that delivery complications can be hazardous to their health, and 62.5% of them agreed that if they get a skilled attendant during delivery, it will be beneficial to their health and the health of their newborns. Regarding their attitude toward institutional delivery, 73.7% of study participants had a positive attitude toward institutional delivery and 26.3% had a negative attitude (Table 3).

### 3.5. Place of Delivery Practice

From 240 women who have been interviewed on a place of delivery, 71.3% of them have delivered in health facilities, while the rest 28.7% have delivered at home. About 60.8% of labor was attended by traditional birth attendants among women who gave birth at home. Fifteen (6.3%) of women faced any type of danger sign during labor and delivery during their last pregnancy and delivery, and eleven of them experienced antepartum hemorrhage. One hundred twenty-six (52.5%) women completed labor in less than a half-day or night (Table 4).

### 3.6. Reason for Home Delivery

**Figure 2:**
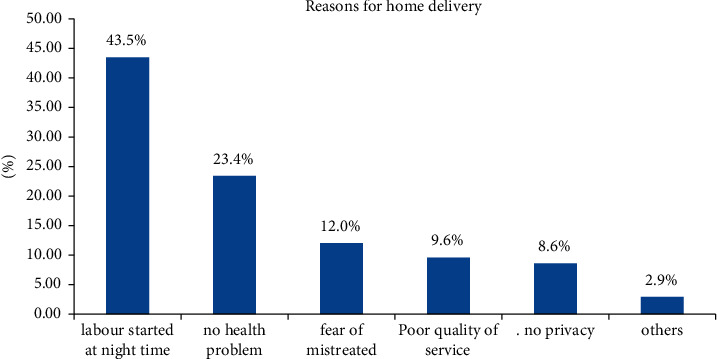
Reason for home delivery among women who gave birth in the last one year at Kersa Woreda, Southwest Ethiopia, July 2021.

Of those women who gave birth at home, about 43.5% mentioned that their reason was that the labor began at night and that they (expectant women) could not walk to the health facility to give birth in the middle of the night. Another major factor was that there were no health issues related to delivery at the health facility (23.4%) (Figure 2).

## 4. Factors Associated with Home Delivery

A bivariate analysis result revealed that women's educational status, monthly income, large family size, decision-making, attitude toward delivery service, knowledge on delivery service, ANC follow-up, women's occupational status, and husbands' educational status were the identified predictors of home delivery. At multivariable logistic regression analysis, monthly incomes, large family size, decision-making, ANC follow-up, attitude toward delivery service, and knowledge on delivery service were the identified predictors of home delivery.

Regarding the monthly income status, women who had lower monthly incomes were 16.8 times more likely to give birth at home as compared to women with higher incomes (AOR = 16.8, 95% CI: (2.028–13.8)). The other predictor of home delivery was a history of ANC follow-up. Study participants who had no history of ANC follow-up were 5.7 times more likely to give birth at home as compared to women who had ANC follow-up (AOR = 5.7, 95% CI: (2.358–16.262)). Concerning the decision-making process for delivery decisions, women whose husbands made the delivery decisions were 5 times more likely to give birth at home as compared to those who jointly made delivery decisions (AOR = 5.0, 95% CI: (1.252–20.021)). Women with large family sizes were 2.2 times more likely women to deliver at home that women with small family sizes ((AOR = 2.2, 95% CI: (1.187–4,119)).

The knowledge of women toward delivery service was another predictor of home delivery. Those women who had poor knowledge of delivery services were 3 times more likely to give birth at home as compared to those women who had good knowledge (AOR = 3.0, 95% CI: (1.661–5.393)). Women who had a negative attitude toward delivery services were 2.2 times more likely to give birth at home as compared to those who had a positive attitude (AOR = 2.2, 95% CI: (1.054–4.409)) (Table 5).

## 5. Discussion

In this study, the extent of home delivery was found to be 28.7%. This study is almost similar to the findings in Debremarkos; 25.3% of women were delivered at home [17]. However, the present finding was lower than studies conducted in Anlemo district, Southern Ethiopia (49.3%) of women delivered at home; in Fogera district, Northwest Ethiopia (68.4%) of women delivered at home; and in Zala Woreda, Southern Ethiopia, 67.6% of women delivered at home, and a meta-analysis in Ethiopia showed home delivery at 48.5% [13,14,18,19]. The possible reasons for the discrepancy could be explained in part by variations in infrastructure availability for easy access to health care facilities, geographic location, and the time interval between studies of population surveys.

Home delivery was found to be strongly linked to income. Women with low monthly incomes were 16.8 times more likely than rich women to give birth at home. This finding is similar with other studies conducted in rural Ayacucho and Ethiopia [9,20]. Even though maternal health care is free, transportation expenses vary, and they are required to buy some materials for delivery that are not available at the health facility.

In this study, ANC follow-up was another predictor of home delivery. Those women who had never received ANC follow-up in their last pregnancy were 5.7 times more likely to give birth at home as compared to women who had ANC follow-up. Similar findings were reported in the study performed at Zala Woreda [20]. This could be because women who attended ANC visits learned about the necessity of institutional birth and the maternal and newborn complications that can occur during delivery and the postnatal period.

In this study, the decision-making power of the women was another predictor of the place of delivery. Women whose husbands made the delivery decision were 5 times more likely to give birth at home as compared to those who jointly made delivery service decisions. This was similar to the findings from Eritrea [21]. This might be because decision-making power concerning the use of maternal healthcare services is strongly influenced by these values. Joint decision-making enables evidence and women's preferences to be incorporated into a consultation, improving their knowledge on delivery service, risk perception accuracy, and women-healthcare providers' communication, reducing decision conflict and enhancing institutional delivery service that reduces maternal mortality and morbidity related to home delivery.

In this study, family size was also a predictor of home delivery. Those women with large family sizes were 2.2 times more likely to give birth at home than women with small family sizes. This corresponds to the findings of a study carried out in Rwanda [15]. This may be because women with large family sizes have no money to seek out a health facility for delivery and may not give value to the importance of institutional delivery. Women who had poor knowledge of delivery services were 3 times more likely to give birth at home as compared to women who had good knowledge of delivery services; it is similar to a study done in rural Bangladesh [22]. This is due to knowledge that can provide the women the advantage of health facility delivery for both the women and their newborns. The attitude of women toward delivery services was another predictor of home delivery. Women who had a negative attitude toward delivery services were 2.2 times more likely to give birth at home as compared to those who had a positive attitude. This is similar to study findings in the Amhara and Mongomo regions of Guinea Equatorial [16,23]. This could be because women who have a negative attitude toward delivery services are not likely to attend antenatal care, and as such, will have poor knowledge toward delivery services and will not value health facility delivery.

The reliability of the data was maintained as the study was a community-based survey, which was one of the study's strength. However, since the study was a cross-sectional study, the possibility of recall bias may result in underreporting of the results.

## 6. Conclusion

In this study, the extent of home delivery among women who gave birth in the last year was low when compared with EDHS 2016. The identified factors associated with home delivery were low monthly income, only the husband as the decision-maker, no antenatal care follow-up, poor knowledge toward delivery services, negative attitude toward delivery services, and large family size. Healthcare professionals and health extension workers should give awareness on institutional delivery and on birth preparedness to deliver at health facilities even at the sudden onset of labor.

## Figures and Tables

**Table 1:** Sociodemographic characteristics of women who gave birth in the last one year at Serbo, Kersa Woreda, Southwest Ethiopia, July 2021.

Variables	Response	Frequency	Percentage
Age (years)	20–24	68	28.3
	25–29	111	46.3
	30–34	44	18.3
	35–39	17	7.1

Marital status	Married	223	92.9
	Divorced	11	4.6
	Widowed	6	2.6

Religion	Muslim	142	59.2
	Protestant	40	16.6
	Orthodox	58	24.2

Mother's occupation	House wife	115	47.9
	Merchant	97	40.4
	Farmer	14	5.8
	Government employee	14	5.8

Mother's educational status	Illiterate	87	36.3
	Primary education	133	55.4
	Secondary and above education	20	8.3

Monthly income	Low	46	19.2
	Medium	158	65.8
	High	36	15.0

Husband's educational status	Illiterate	7	2.9
	Read and write	96	40
	Primary education	79	32.9
	Secondary and above education	58	24.2

Monthly income	Low	46	19.2
	Medium	158	65.8
	High	36	15.0

**Table 2:** Obstetric characteristics of women who gave birth in the last one year at Serbo, Kersa Woreda, Southwest Ethiopia, July 2021.

Variables	Categories	Frequency	Percent
Age of mother at first pregnancy	16–20	121	50.4
	21–26	119	49.6

Gravida	Primigravida	169	70.4
	Multi gravid	71	29.6

Parity	1–2	177	73.8
	≥3	63	26.2

Wanted pregnancy	Yes	195	81.3
	No	45	18.8

Attendee ANC in last pregnancy	Yes	179	74.6
	No	61	25.4

Place attended for ANC follow-up	Hospital	6	3.4
	Health center	173	96.6

^*∗*^advantages of ANC follow-up	To assess maternal health conditions	170	94.3
	To assess the fetal health condition	162	90.5
	To assess fetal lying	121	67.5
	To anticipate possible delivery complications	66	36.9
	I do not know the advantages	58	32.4

Time when ANC follow-up started	First trimester	165	92.2
	Second trimester	14	7.8

Number of ANC follow-ups attended during the last pregnancy	1	3	1.7
	2	103	57.5
	3	67	37.4
	≥4	6	3.4

Reasons for not attending ANC	No health problem	26	42.6
	Too busy to attend ANC	24	39.3
	Others	11	18.1

Faced any danger signs during your last pregnancy	Yes	25	10.4
	No	215	89.6

Type of danger signs you have faced	Vaginal bleeding	11	44
	Headache	9	36
	Others	5	20

Received any counsel regarding place of delivery	Yes	160	91.1
	No	16	8.9

Receive any counsel regarding possible pregnancy and delivery complications	Yes	160	91.1
	No	16	8.9

^*∗*^Multiple responses.

**Table 3:** Attitude toward institutional delivery among women who gave birth in the last one year at Serbo, Kersa Woreda, Southwest Ethiopia, July 2021.

Variables	Response	Frequency	Percent
Any pregnant woman is susceptible to face delivery complications	Agree	150	62.5
	Neutral	50	20.8
	Disagree	40	16.7

Like any pregnant woman, I am susceptible to face delivery complications	Agree	160	66.7
	Neutral	50	20.8
	Disagree	30	12.5

Delivery complications can be severe and may be hazardous to my wellbeing	Agree	150	62.5
	Neutral	50	20.8
	Disagree	40	16.7

Delivery at home can be severe and may be hazardous to the newborn	Agree	160	66.7
	Neutral	50	20.8
	Disagree	30	12.5

Being attended by a skilled delivery attendant may be beneficial to the newborn and wellbeing	Agree	150	62.5
	Neutral	50	20.8
	Disagree	40	16.7%

Delivery at a health facility may shorten the duration of labor	Agree	150	62.5
	Neutral	50	20.8
	Disagree	40	16.7

Being delivered at a health facility helps me to get health information to prevent postpartum complications for me and my newborn	Agree	150	62.5
	Neutral	50	20.8
	Disagree	40	16.7

Delivery at a health facility causes loss of privacy	Agree	160	66.7
	Neutral	50	20.8
	Disagree	30	12.5

I could not buy the items, so I did not go to delivery at the health facility	Agree	160	66.7
	Neutral	50	20.8
	Disagree	30	12.5

If you deliver at home, your women and husbands will assist you to deliver safely	Agree	150	62.5
	Neutral	50	20.8
	Disagree	40	16.7

Over all attitude category	Positive attitude	177	73.7
	Negative attitude	63	26.3

**Table 4:** Place of delivery practice among women who gave birth in the last one year at Serbo, Kersa Woreda, Southwest Ethiopia, July 2021.

Variables	Response	Frequency	Percent
Place of delivery in last pregnancy	Health institution	171	71.3
	Home	69	28.7

Delivery assisted if at home delivery	TBA	42	60.8
	Relative	3	4.2
	Mother	22	32
	Neighbor	2	3

Faced any health problems during labor and delivery	Yes	15	6.3
	No	225	93.7

^*∗*^Types of health problems during labor and delivery	APH	11	44.0
	Elevated blood pressure	8	32.0
	Others	6	24.0

Duration of labor	Less than half day or night	126	52.5
	One day or one night	103	42.9
	More than one day or one night	11	4.6

Any health problem of the alive baby	Yes	7	2.9
	No	233	97.1

Decision maker for the place of delivery	Self	154	64.2
	Husband	13	5.4
	Jointly	73	30.4

^*∗*^Multiple responses.

**Table 5:** Multivariate logistic regression analysis for home delivery at Serbo, Kersa Woreda, Jimma Zone, Southwest Ethiopia, 2021.

Variables	Category	Place of delivery		COR (CI 95%)	AOR (CI 95%)	*p* value
		Home	Institutional			
Monthly income	Low	18 (26.1%)	28 (16.4%)	22.5 (2.828–17.9)^*∗*^	**16.8 (2.028–13.8)** ^*∗∗*^	0.009
	Medium	50 (72.5%)	108 (63.2%)	16.2 (2.158–121.642)^*∗*^	15.3 (.981–11.78)	0.069
	High	1 (1.4%)	35 (20.5%)	1	1	

Family size	Small	40 (58%)	131 (76.6%)	1	1	0.012
	Large	29 (42%)	40 (23.4%)	2.4 (1.310–4.304)^*∗*^	**2.2 (1.187–4.119)** ^**∗∗**^	

ANC follow-up	Yes	20 (29%)	158 (93%)	1	1	0.00001
	No	49 (71%)	13 (7%)	31.4 (14.82–71.10)^*∗*^	**5.7 (2.358–16.262)** ^**∗∗**^	

Decision maker for the place of delivery	Self	57 (82.6%)	97 (56.7%)	5.4 (2.38–12.90)^*∗*^	5.3 (.28–12.53)	0.072
	Husband	5(7.2%)	8 (4.7%)	5.9 (1.509–23.009)^*∗*^	**5.0 (1.252–20.021)** ^*∗∗*^	0.023
	Jointly	7 (10.2%)	66 (38.6%)	1	1	

Knowledge toward delivery services	Good	28 (40.6%)	111 (64.9%)	1	1	0.0001
	Poor	41 (59.4%)	60 (35.1%)	2.7 (1.53–4.81)^*∗*^	**3.0 (1.661–5.393)** ^*∗∗*^	

Attitude toward delivery services	Positive attitude	56 (81.2%)	121 (70.8%)	1	1	0.035
	Negative attitude	13 (18.8%)	50 (29.2%)	1.8 (0.895–3.540)^*∗*^	**2.2 (1.054–4.409)** ^*∗∗*^	

^*∗*^(*P* < 0.25) in bivariate, 1 = reference group, ^*∗∗*^ statically significant in multivariate. Bold values indicated the statically significant variables.

## Data Availability

Data supporting this study are available from the corresponding author upon reasonable request.

## Abbreviations

ANC: Antenatal care
AOR: Adjusted odds ratio
COR: Crude odds ratio
DHS: Demographic health survey
MMR: Maternal mortality ratio
NGO: Nongovernmental organization
PPH: Postpartum hemorrhageSPSS:
SD: Standard deviation
TBA: Traditional birth attendant
WHO: World Health Organization.

## Additional Points

Obstetric hemorrhage, in which a woman bleeds profusely shortly after giving birth, is the leading cause of maternal mortality (deaths from pregnancy and childbirth-related complications). It usually occurs after a home delivery and is caused by either mismanagement of the second or third stages of labor. Childbirth in a nonclinical setting, such as at home rather than in a health facility, is known as home delivery. The impact of home birth, particularly unattended births, is not confined to maternal health issues; it also leads to perinatal and neonatal morbidity and mortality. Only a small fraction of reproductive-age women in Ethiopia seek professional help during pregnancy and childbirth. If a home delivery is not performed by a qualified professional, the risk of infection, postpartum hemorrhage (PPH), and HIV/AIDS transmission to family members or traditional birth attendants who do not wear protective equipment increases. The extent of home delivery was 28.7%. Health professionals and health extension workers should raise awareness about institutional delivery and birth readiness so that women can give birth at a health facility even if labor begins unexpectedly.
